# Recovery and Return to Activity 1 Year After Ankle Fracture Managed With Open Reduction and Internal Fixation: A Prospective Longitudinal Cohort Study

**DOI:** 10.1177/24730114221091806

**Published:** 2022-04-19

**Authors:** Ailar Ramadi, Lauren A. Beaupre, Luke Heinrichs, M. Elizabeth Pedersen

**Affiliations:** 1 Department of Physical Therapy, Faculty of Rehabilitation Medicine, University of Alberta, Edmonton, Alberta, Canada; 2 Division of Orthopaedic Surgery, Department of Surgery, Faculty of Medicine and Dentistry, University of Alberta, Edmonton, Alberta, Canada

**Keywords:** ankle fracture, open reduction and internal fixation, range of motion, patient-reported outcome measure, return to activity

## Abstract

**Background:**

Ankle fractures are common and frequently managed with open reduction and internal fixation (ORIF). Although these fractures can occur at any age, they are most common in younger individuals with high-energy trauma or older adults with lower-energy trauma. Our investigation focused on those aged 65 years or younger. Our primary objective was to describe recovery over the first postfracture year in (1) functional dorsiflexion using the weightbearing lunge test (WBLT), (2) patient-reported functional outcomes using the Olerud Molander Ankle Score (OMAS), (3) return to prefracture activity levels, and (4) return to work. Secondarily, we examined patient and clinical factors (including the WBLT and OMAS) associated with return to prefracture activities, including sports.

**Methods:**

Using a prospective inception cohort of 142 patients between 18 and 65 years old who underwent ORIF after ankle fracture and attended follow-up visits, we collected information from participants and their medical charts. We assessed functional dorsiflexion (using side-to-side difference in WBLT), patient-reported functional outcome (OMAS), and self-reported return to prefracture activity levels and work at 6 weeks, 6 months and 1 year postoperatively.

**Results:**

The WBLT, OMAS, and return to prefracture activity and work improved significantly over time (*P* < .001). However, at 1 year postoperation, the mean side-to-side difference in the WBLT was 3.22±2.68 cm, 69 (72%) reported ankle stiffness, and only 49 (52%) had returned to prefracture activity levels. Of those who were working, 97% had returned to work by 1 year postoperation. Only the OMAS (*P* < .001) and side-to-side difference in WBLT (*P* = .011) were significantly associated with return to prefracture activity levels.

**Conclusion:**

Although participants improved significantly over the first postoperative year in all outcomes, many reported limitations in functional dorsiflexion and return to prefracture activities. Those with higher OMAS scores and smaller side-to-side difference in WBLT were more likely to return to prefracture activity levels by 1 year postoperatively.

**Level of Evidence:** Level II, prognostic study.

## Introduction

Ankle fractures are common and frequently managed using open reduction and internal fixation (ORIF).^20,23^ Although full return to prefracture activities is generally anticipated, patients often report ongoing pain and stiffness that may restrict their activities or limit their recovery.^1,2^

Studies evaluating recovery after ankle fracture have used a variety of outcomes.^12,15^ Earlier studies of recovery often examined ankle range of motion (ROM) in a nonweightbearing position that does not reflect the functional loadbearing requirement of the ankle.^12^ More recent studies have emphasized other outcomes such as patient-reported outcome measures^15^ or gait^17^ to determine recovery after an ankle fracture, but have found that these measures do not fully explain recovery from fracture.^10^

The weightbearing lunge test (WBLT), commonly used in chronic ankle stability populations,^19^ assesses weightbearing dorsiflexion.^5^ Normative data suggest that a side-to-side WBLT difference of greater than 1.5 cm likely indicates functional ankle impairment.^8^ Measuring weightbearing ankle dorsiflexion (ie, functional dorsiflexion) and using side-to-side difference in the WBLT may inform understanding of patients’ overall recovery after ankle fracture. Further, although return to prefracture activity levels appears to be an important outcome following ankle fracture,^13,16^ limited studies have investigated clinical factors associated with this outcome following an ankle fracture managed by ORIF.^6,9^

Although ankle fractures can occur at any age, they are most common in younger males with more high-energy trauma or older females with low-energy trauma, such as a fall from a standing height.^3^ Because we were interested in return to prefracture activities including sports, the focus of this evaluation was those who were 18-65 years old.

Our primary objective was to describe recovery over the first postoperative year after ankle fracture managed with ORIF in terms of (1) the side-to-side difference in the WBLT to evaluate functional dorsiflexion; (2) the Olerud Molander Ankle Score (OMAS) to evaluate patient-reported outcomes; (3) return to prefracture activity levels, including sports; and (4) return to work (for those who were working at the time of fracture). Secondarily, we examined the association of demographics, baseline clinical and fracture characteristics, and side-to-side difference in the WBLT and OMAS with return to prefracture activity over the first postoperative year.

## Methods

### Study Design

This study was a prospective inception cohort with longitudinal follow-up to 1 year postsurgery of eligible patients who sustained an ankle fracture between January 2014 and December 2016 and were treated at a single tertiary health care center in Edmonton, Canada. Participants were identified and enrolled at the time of surgery and followed over 1 year postoperatively. All participants provided signed informed consent and the study was approved by the Health Research Ethics Board (Pro00041862). During the recruitment period, 260 eligible patients were admitted to hospital and approached to participate. Of these, 155 (60%) participants provided written consent. However, 13 (8%) withdrew prior to any postoperative assessment; thus, our study evaluated 142 participants with postoperative data.

#### Inclusion criteria

Eligible participants were 18-65 years old and underwent ORIF for a uni-, bi-, or trimalleolar ankle fracture with/without associated syndesmotic injury or ankle dislocation.

#### Exclusion criteria

Patients with (1) pilon, plafond, bilateral fractures, or who (2) experienced polytrauma, (3) were nonambulatory prefracture, (4) had cognitive impairment, or (5) did not speak English were excluded.

#### Operative and rehabilitation protocol

As we were interested in the outcomes associated with delivery of usual care to those experiencing an ankle fracture and undergoing ORIF, the surgeons (n = 11) chose the operative approach and fixation at their discretion to manage the fracture. ORIF procedures included plate fixation, screws only, or external fixation. All surgeons were either fellowship-trained foot and ankle surgeons (n = 4) or fellowship-trained trauma surgeons (n = 7) with at least 5 years of clinical experience.

Following surgery, surgeons followed their usual practice for mobility and weightbearing with most patients placed into removable splints or boots to allow early mobility (ie, starting at 2 weeks postoperatively). Weightbearing typically started between 2 and 6 weeks postoperatively.

#### Procedure

The research assistant screened new admissions to the orthopaedic service to identify patients with an ankle fracture. The surgeons reviewed the potential participant’s radiographs to determine if the fracture met the inclusion criteria. Eligible patients were approached by the research assistant independent of the surgeon and informed consent was obtained from willing volunteers.

Baseline information was then collected by the research assistant through patient self-report and chart review. History of any associated medical conditions was also noted using a standardized comorbidity listing. Fracture and operative information including number of malleoli involved, presence of associated dislocation, as well as type of fixation, use of a syndesmotic screw, postoperative immobilization, and weightbearing status were collected by a senior orthopaedic resident. Participants were also asked about their prefracture activity level and employment outside the home and self-selected the physical work level of their employment (Sedentary [eg, office work, retail], light labor [eg, nursing], moderate labor [eg, truck-driving], and heavy labor [eg, construction]), if employed.

Participants were asked to return at 6 weeks, 6 months, and 1 year postoperatively to assess ankle ROM using the WBLT and complete the OMAS measure. At these time points, the research assistant also asked about return to prefracture activity and work.

### Outcome Measures

#### Weightbearing Lunge Test

The WBLT^5^ has good intrarater and interrater reliability in both healthy subjects^5,11^ and following an ankle fracture^22^ and a unilateral minimal detectable change (MDC) of 1.9 cm within and 1.6 cm between raters.^19^ Further, normative data suggest that a side-to-side WBLT difference of greater than 1.5 cm likely indicates functional ankle impairment.^8^

The WBLT was performed by asking the patient to place his or her foot perpendicular to a wall and to lunge his or her knee toward the wall. The subject was able to use the wall for support as required. The foot was moved away until the maximum ankle dorsiflexion was reached (ie, when the heel started to lift off the ground). The distance of the tip of the great toe from the wall was measured in centimeters.^5^ In subjects with limited ankle movement, where the knee could not reach the wall, the distance between the knee and the wall was measured and recorded as a negative number.^22^ The WBLT was completed on both affected and unaffected sides. Side-to-side difference in WBLT (“unaffected” minus “affected” side WBLT score) was then calculated.

#### Olerud Molander Ankle Score

The OMAS is based on the subjective complaints of pain, stiffness, swelling (symptoms subscale) and functional activities of stair climbing, running, jumping, squatting, use of supports, and work/usual activity restrictions (function subscale),^18^ and measures the construct of patient-reported ankle function.^14^ The total score is a 100-point scale, with 100 indicating no ankle symptoms/limitations; the estimated minimally important change is 9.7 points.^14^

#### Return to prefracture activity levels

Return to prefracture activity levels was assessed by asking the participants whether or not they were able to return to their preferred activities, including sports, at prefracture levels and was recorded as a dichotomous variable (yes/no).

#### Return to work

Return to work was assessed by asking the subsample of the participants who were employed prior to fracture (n = 105) whether or not they were able to return to work. We recorded both modified and full return to work, but focused our analysis at 6 months and 1 year on return to prefracture work levels (yes/no).

### Sample Size Calculation

The sample size was based on the expected proportion of patients reporting stiffness at the end of 1 year, which we hypothesized would be associated with return to prefracture activity levels. Based on previous work,^4,7,21^ we expected that at least 25% of participants would report stiffness and planned to enroll 200 participants so that we would have 50 participants reporting stiffness (and likely reduced return to prefracture activities) at the completion of the study. This would give adequate power to consider at least 5 variables that could be associated with return to prefracture activity. Enrollment was truncated at 155 participants as recruitment had continued for 3 years and we had used all allocated resources for the study. However, return to prefracture activity levels was substantially lower than anticipated at 1 year postoperatively. This allowed us to perform our analyses as planned.

### Statistical Analysis

Baseline demographic and clinical characteristics data are presented as mean ± standard deviation (SD) or absolute number (percentage) (Table 1). We assessed change over time in the side-to-side difference in the WBLT and the OMAS (continuous dependent variables) using generalized linear mixed modeling. The changes in the proportion of participants who returned to activities at prefracture levels or to work over time (categorical variables) were assessed using generalized estimating equations (GEEs). Both generalized linear mixed modeling and GEE assess the overall impact of time using all available data, so that data from participants missing a postoperative evaluation could be included.

**Table 1.** Demographic and Clinical Characteristics of 142 Participants With Follow-up Data.

Characteristic	Mean ± SD or n (%)
Age at surgery, mean ± SD	42.8 ± 14.3
Sex	
Female	86 (60.6)
Male	56 (39.4)
Number of comorbidities	
0-1	95 (66.9)
≥2	47 (33.1)
Associated dislocation	
No	91 (64.1)
Yes	51 (35.9)
Use of syndesmosis screw	
No	95 (66.9)
Yes	47 (33.1)
Number of malleoli	
Single malleolar	66 (46.5)
Lateral	61
Medial	5
Bimalleolor	26 (18.3)
Trimalleolar	50 (35.2)
Fixation type	
Plate	126 (88.7)
Screws only	14 (9.9)
External fixator	2 (1.4)
Immobilization type^a^	
Walking boot	109 (76.8)
Cast/half-cast/tensor	27 (19.0)
Not specified	6 (4.2)
Employment physical level (n = 105)	
Moderate/heavy	25 (23.8)
Light/sedentary	80 (76.2)

Data are presented as mean ± SD or as the absolute number (percentage).

a n = 99 of those with walking boots were discharged nonweightbearing whereas n = 3 were feather-weightbearing, n = 3 were partial weightbearing, and n = 1 was weightbearing as tolerated at hospital discharge. All participants who received other immobilization were nonweightbearing for the first 6 postoperative weeks.

Univariate GEE analysis was used to test the association of demographic and clinical characteristics with return to activity over time. Factors associated with return to prefracture activity levels over time were identified using the multivariate GEE analysis. Multivariate GEE analysis was performed including adjusting for age and sex. All statistical tests with a *P* <.05 were considered significant. All analyses were conducted using SAS (SAS institute, version 9.4).

## Results

Of 142 participants, 136 (96%) completed the 6-week assessment whereas 107 (75%) and 94 (66%) completed the 6-month and 1-year follow-up assessments, respectively. Although our selection criteria allowed for surgeons to provide usual care, 141 (99%) participants received early mobilization (ie, after 2 weeks) whereas WBAT was typically started between 2 and 6 weeks postoperatively (n = 118; 87%) based on surgeon preference and fracture pattern. Baseline demographic and clinical characteristics data are presented in Table 1.

On study entry, those who did not return for follow-up visits (n = 13/155) were similar to those who completed the 1-year follow-up assessment in most characteristics (*P* > .11), but of those who withdrew, more were males (*P* = .04) and worked in more physically demanding jobs (*P* = .04) than those who returned for follow-up.

### Weightbearing Lunge Test

The side-to-side difference in the WBLT reduced significantly over the 1-year follow-up (*P* < .001). However, the mean side-to-side WBLT difference was still 3.2 ± 2.7 cm at 1 year postoperatively (Table 2).

**Table 2.** Weightbearing Lunge Test, Olerud Molander Ankle Score, Return to Activity, and Return to Work Over Time.^a^

	6 wk	6 mo	1 y	*P*
Affected WBLT (cm)	−3.27 ± 4.68	4.15 ± 4.14	5.64 ± 3.57	<.001
WBLT side-to-side difference (cm)	9.59 ± 4.89	4.7 ± 3.8	3.22 ± 2.68	<.001
OMAS total	39.01 ± 14.37	68.5 ± 19.66	77.55 ± 16.24	<.001
OMAS stiffness question (yes)	120 (88.9)	88 (82.2)	69 (73.4)	.011
Return to activity (yes)	1 (0.7)	41 (38.7)	49 (52.1)	<.001
Return to work (yes)	51 (50.5) (n = 101)	74 (91.4) (n = 81)	68 (97.1) (n = 70)	<.001

Abbreviations: OMAS, Olerud Molander Ankle Score; WBLT, Weightbearing Lunge Test.

a Data are presented as mean ± SD or as the absolute number (percentage).

### Olerud Molander Ankle Score

Similarly, the OMAS improved over time (*P* < .001). However, 69 of 95 (73%) respondents reported that their ankle remained stiff at 1 year after surgery based on their response to the single OMAS item asking about stiffness (Table 2).

### Return to prefracture activity levels

At 6 weeks after ankle fracture, as expected based on fracture healing, less than 1% of participants had returned to preferred activities at prefracture levels. The proportion of patients who had returned to prefracture activity levels at 6 months was 39% (42/107), and this increased to 52% at the 1-year follow up (*P* < .001) (Table 2).

### Return to work

In the subsample of the participants who were employed prior to fracture (n = 105 [73.9%]), the proportion of participants who returned to work increased significantly over the course of 1 year postoperatively (*P* < .001) (Table 2). At 6 weeks postoperatively, few participants had returned to work, but by 6 months, >90% had returned to work, which increased to >97% by 1 year postoperatively (Table 2).

### Factors associated with return to prefracture activity levels

The results from univariate GEE analysis showed that the clinical characteristics such as fewer comorbidities (*P* = .005), fewer malleoli involved (*P* = .01), no associated dislocation (*P* < .001), and type of fixation (*P* = .02) as well as higher OMAS score (*P* < .001) and lower side-to-side difference in WBLT (*P* < .001) were associated with increased likelihood of return to prefracture activity levels within 1 year of surgery (Table 3).

**Table 3.** Univariate Association of Demographic, Clinical Characteristics and Patient-Reported Outcomes With Return to Activity (RTA) Over Time. ^a^

	Return to Activity						
	6-wk		6 mo		1 y		
	No	Yes	No	Yes	No	Yes	*P*
Age at surgery, y	42.9±14.1	43	42.8±13.6	42.9±15	45.6±13	41.2±14.3	.43
Sex							
Female	82 (100)	0 (0.0)	42 (64.6)	23 (35.4)	29 (49.2)	30 (50.8)	.41
Male	51 (98.2)	1 (1.8)	23 (56.1)	18 (43.1)	16 (45.7)	19 (54.3)	
Number of comorbidities							
0-1	89 (98.9)	1 (1.2)	36 (52.9)	32 (47.1)	24 (39.3)	37 (60.7)	.005
≥2	44 (100)	0 (0.0)	29 (76.3)	9 (23.7)	21 (63.6)	12 (36.4)	
Associated dislocation							
No	76 (98.7)	1 (1.3)	30 (48.4)	32 (51.6)	17 (32.7)	35 (67.3)	<.001
Yes	46 (100.0)	0 (0.0)	26 (78.8)	7 (21.2)	21 (65.6)	11 (34.4)	
Use of syndesmosis screw							
No	85 (98.8)	1 (1.2)	38 (57.6)	28 (42.4)	25 (40.9)	36 (59.1)	.059
Yes	43 (100.0)	0 (0.0)	23 (67.6)	11 (32.4)	18 (64.3)	10 (35.7)	
Number of malleoli							
Single	58 (98.3)	1 (1.7)	24 (51.1)	23 (48.9)	16 (39.0)	25 (61.0)	.01
Bimalleolor	24 (100.0)	0 (0.0)	11 (55.0)	9 (45.0)	8 (50.0)	8 (50.0)	
Trimalleolar	48 (100.0)	0 (0.0)	28 (77.8)	8 (22.2)	20 (57.1)	15 (42.8)	
Fixation type							
Screws	12 (100)	0 (0.0)	9 (81.8)	2 (18.2)	9 (90.0)	1 (10.0)	.02
Screws and plate (±external fixator)^b^	118 (99.2)	1 (0.8)	54 (58.7)	38 (41.3)	35 (42.7)	47 (57.3)	
Immobilization type							
Boot	104 (99.0)	1 (1.0)	51 (62.2)	31 (37.8)	33 (45.8)	39 (54.2)	.75
Cast/half-cast/tensor	26 (100.0)	0 (0.0)	12 (60.0)	8 (40.0)	10 (52.6)	9 (47.4)	
OMAS Total (Post-fracture)	38.68±14.3	65.00±0.00	59.85±17.41	81.95±15.12	67.22±15.58	87.04±9.84	<.001
WBLT side-to-side difference	9.73±4.86	3.00±0.00	5.72±4.10	3.18±2.65	4.14±2.73	2.40±2.38	<.001

Abbreviations: OMAS, Olerud Molander Ankle Score; WBLT, weightbearing lunge test.

a Data are presented as mean ± SD or as the absolute number (percentage).

Univariate generalized estimating equation (GEE) analysis was used.

*P* <.05 was considered significant.

b Only 2 participants received external fixation.

When entered into a multivariate model that also adjusted for age and sex, only a higher OMAS (odds ratio 1.115, 95% CI 1.06, 1.17) and lower side-to side difference in WBLT (odds ratio 0.86, 95% CI .75, .98) were significantly associated with return to prefracture activity levels (Table 4).

**Table 4.** Adjusted Model of Factors Associated With Return to Activity Over Time.^a^

Parameter	Estimate	SE	OR	95% CI		*P*
				Lower Limit	Upper Limit	
Intercept	−9.141	1.986				<.001
Age at surgery	0.013	0.020	1.013	0.974	1.054	.51
Sex (male)^b^	−0.549	0.585	0.577	0.184	1.816	.35
Number of comorbidities (≥2)^c^	−0.190	0.613	0.827	0.249	2.749	.76
Associated dislocation (yes)^d^	−0.969	0.647	0.380	0.107	1.347	.13
Syndesmosis screw (yes)^e^	−0.478	0.632	0.620	0.180	2.137	.45
Number of malleoli (bimalleolar)^f^	−0.095	0.662	0.909	0.248	3.330	.89
Number of malleoli (single malleolar)	−0.011	0.705	0.989	0.249	3.938	.99
Immobilization (cast/half-cast/tensor)^g^	−0.353	0.575	0.702	0.228	2.167	.54
Post-fracture OMAS total	0.109	0.024	1.115	1.063	1.169	**<.001**
WBLT side-to-side difference	−0.155	0.071	0.856	0.745	0.984	**.03**
Follow-up interval: 1-y^h^	1.712	1.198	5.538	0.530	57.928	.15
Follow up interval: 6-m	2.011	1.185	7.474	0.733	76.218	.09

Abbreviations: OMAS, Olerud Molander Ankle Score; OR, odds ratio; WBLT, weightbearing lunge test.

a Multivariate generalized estimating equation (GEE) analysis was used. *P* <.05 was considered significant.

b Reference group = female.

c Reference group = 0-1.

d Reference group = no.

e Reference group = no.

f Reference group = trimalleolar.

g Reference group = boot.

h Reference group = 6-week.

## Discussion

Patients often indicate ongoing functional limitations related to ankle pain and stiffness after ankle fracture,^1^ but it remains unclear which patient characteristics or clinical factors might be associated with return to prefracture activity levels.^10^ Although significant improvement in the WBLT and OMAS occurred over time, 73% of participants indicated that their ankle was “stiff” at 1 year after surgery. Further, the mean side-to-side WBLT difference was more than 3 cm at 1 year, substantially greater than normative WBLT side-to-side differences.^8^ Thus, the WBLT appears useful to detect functional impairment in those with ankle fracture.

Although most participants who were working prefracture returned to work within 6 months, just more than half had returned to prefracture activity levels at 1 year after their fracture. We were limited in our return to work analysis as most of those who worked prefracture returned to work between 6 weeks and 6 months, limiting our ability to accurately measure this trajectory. We also did not limit our criteria for study participation to those who had physical jobs that required prolonged standing or heavy manual labor, which could also affect the time to return to work as those with sedentary work would be expected to have an earlier return than those with physically demanding jobs. The discrepancy in the proportions of those who returned to work and those who returned to prefracture activity levels was not anticipated. It is possible that return to preferred activities continued to improve beyond the first postfracture year.

Previous studies reported age, gender, comorbidities, number of malleoli involved, presence of syndesmotic injury,^6^ and patient-reported outcomes such as self-reported pain, function, activity limitations, and ability to ambulate as the factors that may influence return to activity after ankle fracture.^9^ When we investigated the multivariate association of demographics, clinical and fracture characteristics, including both the OMAS and WBLT with return to activity, we found that only the OMAS and side-to side difference in WBLT were significantly associated with return to prefracture activity levels over first postoperative year.

The known threshold of side-to-side asymmetry that suggests ankle functional impairment in the WBLT (ie, >1.5 cm between sides) may be a useful tool in the clinic. Detecting functional ankle ROM impairment following ankle fracture may indicate that further rehabilitation should be considered to improve functional movement where possible; further research is needed to determine if this is a useful clinical marker.

To our knowledge, this is the largest series of patients with ankle fracture aged <65 years assessed using the WBLT. Our findings that an ankle fracture can result in limitations in performing prefracture activities, including sports, beyond 1 year postoperatively likely has broad applicability for these patients.

The OMAS is also a useful tool to understand patient perceptions of their functional limitations. Our finding that both the OMAS and side-to side difference in WBLT were independently associated with return to prefracture activity suggests that they are likely measuring different aspects of recovery after ankle fracture and both are useful clinical tools to assess recovery after ankle fracture. Although the OMAS includes a question about return to work and daily activities and requirement to modify job duties, we expanded this evaluation to examine return to prefracture activity levels, including sports. However, it is possible that some participants were responding to both of these questions (OMAS and return to prefracture activities) in a similar fashion.

Despite being one of the largest published series, we were limited in our ability to explore the WBLT and OMAS performance within fracture type. We were evaluating usual care at a large tertiary center with experienced fellowship-trained foot and ankle or trauma orthopaedic surgeons who were able to choose fixation, mobilization, and weightbearing status based on the fracture pattern and their preferred practices. Although there was some heterogeneity across surgeons, most participants were initially nonweightbearing, but still performed early active ankle ROM starting at 2 weeks postoperatively. Weightbearing status typically increased to WBAT between 2 and 6 weeks postfracture.

In addition, because of the large geographical area that this tertiary health centers serves, we had a higher rate of missed assessments over the 1-year assessment period than anticipated, as some participants did not return for all follow-up assessments because of living a substantial distance from the center. Male patients as well as those working in heavier physical jobs were more likely to not have returned for any follow-up assessments; thus, our reported findings may not represent these groups well. Finally, we also only followed participants for 1 year, so improvements in functional dorsiflexion and return to prefracture activity levels were still possible.

## Conclusions

In summary, just over half of participants had returned to prefracture activity levels at 1 year after surgery. Participants with higher postfracture OMAS and smaller side-to-side difference in WBLT were more likely to return to prefracture activity levels within 1 year after surgery than those reporting more asymmetry in ankle ROM and lower self-reported outcomes. Further, the side-to-side difference in WBLT may identify patients who have functional limitations because of restricted weightbearing dorsiflexion that may benefit from further rehabilitation. However, more research is needed to determine if rehabilitation focused on restoring functional dorsiflexion can help patients to return to their prefracture activity levels. We suggest that both patient-reported outcomes and the WBLT be used to assess recovery after ankle fracture.

## References

[1] Beckenkamp PR Lin CW Chagpar S Herbert RD van der Ploeg HP Moseley AM. Prognosis of physical function following ankle fracture: a systematic review with meta-analysis. J Orthop Sports Phys Ther. 2002;44(11):841-851. 10.2519/jospt.2014.5199 25269609

[2] Beckenkamp PR Lin CW Engelen L Moseley AM. Reduced physical activity in people following ankle fractures: a longitudinal study. J Orthop Sports Phys Ther. 2016;46(4):235-242. 26954274 10.2519/jospt.2016.6297

[3] Beerekamp MSH de Muinck Keizer RJO Schep NWL Ubbink DT Panneman MJM Goslings JC . Epidemiology of extremity fractures in the Netherlands. Injury. 2017;48(7):1355-1362. 28487101 10.1016/j.injury.2017.04.047

[4] Belcher GL Radomisli TE Abate JA Stabile LA Trafton PG. Functional outcome analysis of operatively treated malleolar fractures. J Orthop Trauma. 1997;11(2):106-109. 9057145 10.1097/00005131-199702000-00007

[5] Bennell KL Talbot RC Wajswelner H Techovanich W Kelly DH Hall AJ. Intra-rater and inter-rater reliability of a weight-bearing lunge measure of ankle dorsiflexion. Aust J Physiother. 1998;44(3):175-180. 11676731 10.1016/s0004-9514(14)60377-9

[6] Colvin AC Walsh M Koval KJ McLaurin T Tejwani N Egol K. Return to sports following operatively treated ankle fractures. Foot Ankle Int. 2009;30(4):292-296. 19356351 10.3113/FAI.2009.0292

[7] Day GA Swanson CE Hulcombe BG. Operative treatment of ankle fractures: a minimum ten-year follow-up. Foot Ankle Int. 2001;22(2):102-106. 11249218 10.1177/107110070102200204

[8] Hoch MC McKeon PO. Normative range of weight-bearing lunge test performance asymmetry in healthy adults. Man Ther. 2011;16(5):516-519. 21429784 10.1016/j.math.2011.02.012

[9] Johnson JD Chachula LA Bickley RJ Anderson CD Ryan PM. Return to duty following open reduction and internal fixation of unstable ankle fractures in the active duty population. Mil Med. 2019;184(5-6):e381-e384. 10.1093/milmed/usy325 30517675

[10] Keene DJ Vadher K Willett K , et al. Predicting patient-reported and objectively measured functional outcome 6 months after ankle fracture in people aged 60 years or over in the UK: prognostic model development and internal validation. BMJ Open. 2019;9(7):e029813. 10.1136/bmjopen-2019-029813 PMC6661636 31340972

[11] Konor MM Morton S Eckerson JM Grindstaff TL. Reliability of three measures of ankle dorsiflexion range of motion. Int J Sports Phys Ther. 2012;7(3):279-287. 22666642 PMC3362988

[12] Lin CW Donkers NA Refshauge KM Beckenkamp PR Khera K Moseley AM. Rehabilitation for ankle fractures in adults. [Update of Cochrane Database Syst Rev. 2008;(3):CD005595; PMID: 18646131]. Cochrane Database Systematic Rev. 2012;11:CD005595. 10.1002/14651858.CD005595.pub3 23152232

[13] McKeown R Kearney RS Liew ZH Ellard DR. Patient experiences of an ankle fracture and the most important factors in their recovery: a qualitative interview study. BMJ Open. 2020;10(2):e033539. 10.1136/bmjopen-2019-033539 PMC7044932 32024789

[14] McKeown R Parsons H Ellard DR Kearney RS. An evaluation of the measurement properties of the Olerud Molander Ankle Score in adults with an ankle fracture. Physiotherapy. 2021;112:1-8. 34000602 10.1016/j.physio.2021.03.015

[15] McKeown R Rabiu AR Ellard DR Kearney RS. Primary outcome measures used in interventional trials for ankle fractures: a systematic review. BMC Musculoskelet Disord. 2019;20(1):388. 31455297 10.1186/s12891-019-2770-2 PMC6712770

[16] McPhail SM Dunstan J Canning J Haines TP. Life impact of ankle fractures: qualitative analysis of patient and clinician experiences. BMC Musculoskelet Disord. 2012;13:224. 23171034 10.1186/1471-2474-13-224 PMC3517753

[17] Nilsson GM Jonsson K Ekdahl CS Eneroth M. Effects of a training program after surgically treated ankle fracture: a prospective randomised controlled trial. BMC Musculoskelet Disord. 2009;10:118. 19781053 10.1186/1471-2474-10-118 PMC2760502

[18] Olerud C Molander H. A scoring scale for symptom evaluation after ankle fracture. Arch Orthop Traum Surg. 1984;103(3):190-194. 10.1007/BF00435553 6437370

[19] Powden CJ Hoch JM Hoch MC. Reliability and minimal detectable change of the weight-bearing lunge test: a systematic review. Man Ther. 2015;20(4):524-532. 25704110 10.1016/j.math.2015.01.004

[20] Scott LJ Jones T Whitehouse MR Robinson PW Hollingworth W. Exploring trends in admissions and treatment for ankle fractures: a longitudinal cohort study of routinely collected hospital data in England. BMC Health Serv Res. 2020;20(1):811. 32867779 10.1186/s12913-020-05682-9 PMC7457765

[21] Shah NH Sundaram RO Velusamy A Braithwaite IJ. Five-year functional outcome analysis of ankle fracture fixation. Injury. 2007;38(11):1308-1312. 17888434 10.1016/j.injury.2007.06.002

[22] Simondson D Brock K Cotton S. Reliability and smallest real difference of the ankle lunge test post ankle fracture. Man Ther. 2012;17(1):34-38. 21959254 10.1016/j.math.2011.08.004

[23] Vieira CD Dubois-Ferriere V Gamulin A , et al. Operatively treated ankle fractures in Switzerland, 2002-2012: epidemiology and associations between baseline characteristics and fracture types. BMC Musculoskelet Disord. 2021;22(1):266. 33706724 10.1186/s12891-021-04144-5 PMC7953683

